# Error metrics determination in functionally approximated circuits using SAT solvers

**DOI:** 10.1371/journal.pone.0227745

**Published:** 2020-01-14

**Authors:** Sa’ed Abed, Ali A. M. R. Behiry, Imtiaz Ahmad

**Affiliations:** Computer Engineering Department, College of Engineering and Petroleum, Kuwait University, Kuwait, Kuwait; Fayoum University Faculty of Computers and Information, EGYPT

## Abstract

Approximate computing is an emerging design paradigm that offers trade-offs between output accuracy and computation efforts by exploiting some applications’ intrinsic error resiliency. Computation of error metrics is of paramount importance in approximate circuits to measure the degree of approximation. Most of the existing techniques for evaluating error metrics apply simulations which may not be effective for evaluation of large complex designs because of an immense increase in simulation runtime and a decrease in accuracy. To address these deficiencies, we present a novel methodology that employs SAT (Boolean satisfiability) solvers for fast and accurate determination of error metrics specifically for the calculation of an average-case error and the maximum error rate in functionally approximated circuits. The proposed approach identifies the set of all errors producing assignments to gauge the quality of approximate circuits for real-life applications. Additionally, the proposed approach provides a test generation method to facilitate design choices, and acts as an important guide to debug the approximate circuits to discover and locate the errors. The effectiveness of the approach is demonstrated by evaluating the error metrics of several benchmark-approximated adders of different sizes. Experimental results on benchmark circuits show that the proposed SAT-based methodology accurately determines the maximum error rate and an average-case error within acceptable CPU execution time in one go, and further provides a log of error-generating input assignments.

## Introduction

Approximate computing has emerged as a promising architectural concept which offers new opportunities to design circuits or systems which can be more compact, faster and/or consume less power at the cost of a slight loss of accuracy for error-tolerant applications [1]. Error- tolerant applications are the ones where precise computational accuracy is not required which include deep machine learning, image classification, and digital signal processing (DSP) [2]. For example, in DSP applications, human perception is not precise enough to detect a certain range of errors. Therefore, a product of identical (perceived) quality can be presented with lower energy costs or higher performance. Chippa *et al*. [2] estimated that in certain applications, about 83% of runtime is spent on operations that can be approximated. Different types of approximations have been reported in literature for the design of approximated circuits [1]. The form of approximation addressed in this paper is known as functional approximation; in which the Boolean functions implemented into the circuits are inherently different from the original ones with a certain threshold of error between the outputs of both functions.

The emergence of approximate computing has led to the need for adequate evaluation methods for approximate circuits where besides normal metrics such as area, delay, and power consumption, measurement of error metrics is an important consideration and a key challenge. Error metrics provide a quantifiable measure to judge exactly how far off an approximate design is from the correct one, which is critical for the use of approximate circuits in real-life applications. Some of the error metrics measured by existing approaches include worst-case error, average case error, error rate (error probability), and maximum bit-flip error (maximum Hamming distance) [3]. Worst-case error, as the name implies, is the worst possible deviation in values between the two circuits for a single input assignment, which is relevant, for example, in a DSP application where a worst-case error pixel would stand out. Average-case error is the expected error mean value to be found across all input assignments. This would also be seen in a DSP environment where the overall color grading of an approximated image could be affected by a poor average-case error. Maximum bit-flip error is the number of incorrect bits in the approximated circuit, which is relevant to memory address approximation. Error rate is the percentage of input assignments that produce errors in the approximated design that is an indication of the number of introduced errors in the design by the approximation process.

The error metrics mentioned earlier are independent from one another. A circuit with a high error rate does not imply that there is a high maximum bit-flip error or high worst-case error. The relevancy of an error metric is purely based on the application the design is intended for. However, determination of error metrics in approximate circuits is a hard problem [3]. Current error metric evaluation methods suffer from several problems. Simulation-based approaches have prolonged runtimes, and symbolic BDD [4] representation suffers from the state explosion problem. Finally, analytical methods are intractable for larger designs. SAT solver methods [5] have been reported to determine worst-case error and bit-flip error, but not for the maximum error rate or an average-case error, as will be explained in more detail in the related work section. SAT solvers are tools that can determine if a Boolean function is satisfiable; there is a combination of inputs that produces a logic 1.

**Fig 1:**
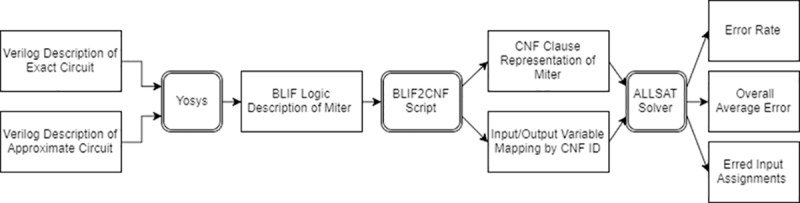
Overview of the proposed methodology.

In this paper, we propose a novel methodology, using SAT solvers to accurately and efficiently determine the maximum error rate and an average-case error. Recent SAT solvers are quick enough to efficiently check relaxed equivalence in many practical situations. We focus on the maximum error rate and an average-case error since these are the hardest to compute, requiring enumeration of all errors and calculation of their values. However, the proposed approach is applicable and can measure all relevant error metrics, since we generate all inputs that produce errors (and their corresponding output values), and then can calculate any of the error metrics. An overview of the proposed methodology is depicted in Fig 1, in which first the approximated and exact designs are combined using an auxiliary circuit called the approximation miter. The approximation miter transforms both designs into one design with a single output and the same inputs. The output of the miter is 0 when the two designs match and are correct. The output of the miter is 1 when the approximated circuit’s output has an erred bit. This new circuit is fed into the SAT solver to determine the error metrics. The proposed evaluation methodology is important since it provides a relatively fast and reliable way for designers to evaluate their approximate circuit designs. The effectiveness of the approach is demonstrated by evaluating the error metrics of several benchmark-approximated adders of different sizes.

The main contributions of this paper can be summarized as follows:

- We propose a SAT-Solver-based algorithm for all error metrics (particularly the maximum error rate and an average-case error) in approximated circuits, given an original circuit.
- The method takes advantage of the computational miter to combine the two circuits and eventually transform the circuit into a CNF format, while saving the original variable mapping to the inputs.
- The algorithm can calculate all error metrics and identify the input configurations that produced them in a single run.
- We prove the correctness of our approach by testing the algorithm on commonly used approximate adders and comparing them with the exact adders.

The remainder of this paper is organized as follows. Various error metrics determination methods applicable to approximate circuits are presented in Section 2. Section 3 provides an overview of the terminology and key concepts used in this work, and describes the error metrics and the main functionality of a SAT solver. The problem statement and proposed solution methodology are discussed in Section 4. A proof of method correctness and an evaluation of the proposed methodology supported by experimental results is described in Section 5. Finally, conclusions and future research opportunities are presented in Section 6.

## Related work

Error metrics are essential tools for of evaluation digital circuits. This is particularly true in the case of approximate circuits, where it is a tool for measuring the adequate correctness of a design that is made to not be precisely exact. The degree of acceptance of approximate designs is usually subjected to many error metrics, such as error rate, maximum bit-flip error, worst-case error, or average-case error.

Typically, even though worst-case errors can be computed for a specific component, on its own, the accumulated worst-case error may differ significantly. Therefore, individual analysis of isolated components is not sufficient for evaluation of approximate circuits. It requires a different set of tools to evaluate the error metrics of larger circuits. Furthermore, different error metrics have varying significance depending on the circuit’s application. Although error-rate may not be a significant metric in some applications, where the error significance/worst-case error may matter more (the value of a pixel), there are several other applications where error-rate is an important metric (e.g. the number of incorrect memory address computations in a microprocessor) [6]. Apart from the application-specific significance of error rate, it is a general quality metric for approximate designs. Error-rate multiplied with error-significance is used as a composite quality metric for approximate circuit designs [7].

There have been many designs and synthesis methods for approximate computing circuits, but not many on precise error metrics [5]. Several approaches determine these metrics, including simulation, analytical analysis, Boolean Satisfiability (SAT solvers), and BDDs. Methods of formal design verifications to evaluate error metrics have been used and implemented, such as Bounded Model Checking (BMC). Currently, the techniques used most often to evaluate the approximate designs are random simulation and error estimation, which are inexact approaches [8, 9]. When BMC is applied to sequential equivalence checking, the circuit is unrolled and the overall problem including the properties is solved using a SAT solver [5]. Some error metrics such as error-rate cannot be expressed in terms of Boolean functions efficiently since it requires counting in the solution space, which is a SAT problem [10].

The authors of [5] proposed a methodology and tool to determine precisely how error behaves in approximated combinational components in sequential circuits. They implemented the use of an approximation miter and preformed model checking using Property-Directed Reachability (PDR). They calculated several error metrics and tried to answer multiple verification questions. In addition, they attempted to determine the earliest time (in clock cycles) that can exceed a certain worst-case error, the maximum case error, and the maximum bit-flip error. They tested their verification package on a number of approximate designs and reported the results. However, they did not calculate metrics for error rate or average-case error.

Yu *et al*. [8] presented a case study to analyze the output quality of imprecise adders for their use in approximate computing. The authors adopted a formal verification of approximate adders based on BDDs rather than random simulation or error estimation in order to retrieve exact error analysis. In order to adapt their methods to the BDD, XOR gates were used to check the equivalence of the outputs followed by a “miter” (a word-wide OR gate). The proposed framework could compute the exact error rates of the designs studied. The methods provided were able to generate test patterns that cover all possible errors produced by the imprecise adders. The work of the authors remained to be tested on larger circuits, such as multiplication. This is an important point, as BDDs suffer from state explosion problems. In [9], Anteneh *et al*. presented an automatic test pattern generation approach for approximate circuits based on Boolean satisfiability. The technique reduced the number of faults and the testing time, while maintain high fault coverage.

Chandrasekharan *et al*. [10] proposed an automatic synthesis methodology for approximating circuits using And-Inverter Graph (AIG) rewriting. The synthesis approach employed bounds on the approximation errors introduced in the design. The methodology was tested on a variety of designs and benchmarks. The authors claimed that their results were comparable with hand-crafted approximate circuits. Their evaluation of error-rate involved using BDDs. The exact approach used was unique to BDD-based representation. This led to extended run-times caused by the need for conversion from AIG to BDD. They also presented an algorithm to calculate bit-flip error using a SAT solver.

Choudhury *et al*. [11] proposed a concurrent error masking methodology based on the use of approximate logic circuits. The approach was estimated to mask 88% of targeted errors for a 34% area overhead and 17% power overhead because of the use of approximate circuits. They evaluated error metrics for their approach via simulation of the approximate logic circuit designs and employed the use of reliability analysis tools.

Jiang *et al*. [12] presented a comparative study between approximate multipliers. The authors provided a review of the different implementations of approximate multipliers’ current designs and compared their evaluations. The authors also used Monte Carlo simulation using MATLAB for error metric determination in their comparison of approximate multipliers. Furthermore, they evaluated the multipliers based on their performance in image sharpening in the MATLAB environment.

Momeni *et al*. [13] proposed designs of approximate compressors to be used in multiplication. Approximate adders were not viable to produce multiplication because of the error accumulation. Previous work had been done on multipliers, but the paper is unique in its use of compressors for multiplication. Two approximate compressors and four approximate multipliers were simulated using HSPICE.

Huang *et al*. [14] provided an overview of imprecise hardware, improved upon imprecise adders, and proposed imprecise multipliers. They stated that numerical analytical analysis was a superior method of error metric computation, when compared to simulation. Their methodology followed two constraints: the only allowed operations were addition and multiplication, and input data were independent of one another. The approach used Interval Arithmetic and Affine Arithmetic for error evaluation. They concluded that their methodology provided higher speed computation with reasonable estimation close to exhaustive search with runtime being orders of magnitude less than Monte Carlo simulation.

Most of the past works used simulation as a means of estimating the error metrics, however, there have been combined methodology that used simulation and other means to maintain accuracy of evaluation and avoid exponentially increasing runtime. Venkatesan *et al*. [15] proposed a methodology for Modeling and Analysis of Circuits for Approximate Computing (MACACO). MACACO used several methods for its formal verification. Worst-case error was computed using a pseudo-SAT solver approach. The authors also computed error probability and average-case error by two means, a BDD package and Monte Carlo-based simulation. The Monte Carlo simulation method was used to assume scalability, as BDD based computation was not deemed feasible for all circuits according to the authors.

Similarly, Soeken *et al*. [16] used BDD-based methods to compute error metrics. This was to their benefit, as their methodology for synthesizing approximate circuit designs was also based on BDD minimization of Boolean functions. Therefore, some of the error metrics (error rate) were calculated during the minimization process. It was stated that because the methodology was based on BDDs, the error metric evaluation was not applicable on much larger circuits in their current implementation. The authors suggested changing the underlying data structure to AIGs; however, it was not clear if error metrics can be computed efficiently.

Recently, Froehlich *et al*. [17] proposed a formal three-stage approach for the determination of all error-metrics for an approximate circuit. The determination of several error-metrics was facilitated by the main bulk of the work being done by a Gröbner reduction and an Algebraic Decision Diagram (ADD) that did not need to be repeated for different error metrics. They used a recursive and hash function to determine the minterms.

Other means of error metric estimation include probabilistic analysis. Mazahir *et al*. [18] proposed a generic methodology for calculating the exact probability of occurrence of any errors in approximate adders and the probability mass function (PMF) of errors for any input distribution without the need of exhaustive or Monte-Carlo simulation. The methodology was based on the internal structures of adders and the probability of carry-in/carry-out. It is worth noting that the proposed approach is specific to approximate adders only, and is applicable when a combination of approximate adders is used. Later, Qureshi *et al*. [19] used HOL4 interactive theorem, proving for probability distribution and error analysis of three high-speed, low-latency approximate adders with uniformly distributed inputs. Mazahir *et al*. [20] provided a probabilistic analysis of errors in an approximate multipliers construct from given blocks. The methodology was based on the internal probabilistic behavior of the building blocks cascading to create larger multipliers. The analysis of [20] was generalized for any input probability distribution and the probability mass function of error was found. The experimental results applied the methodology on state-of-the-art multipliers to compute their probability mass functions and predicted their performance in an image-processing application. The authors of [21] proposed a probabilistic analysis methodology for analyzing two-part segmented adders and derived the mean error distance and mean square error in the approximate adders.

Similarly, Wu *et al*. [22] adopted an analytical methodology with assumptions on the inputs to the approximate circuits. They assumed that inputs were uniformly distributed to adders and thus proposed a method based on the building blocks of approximate adders. The method was also shown to be able to compute the probability mass function of errors (error distribution). It was experimentally shown that the procedure was less computationally extensive than Monte Carlo simulation. It is worth noting that the methods reviewed in [20] and [22] are precise and are suitable only for block-based multipliers and adders, respectively. Furthermore, depending on the application and context a hardware component is used in, it might not necessarily be possible to assume a probability distribution for input assignments.

A popular approach to evaluate approximate circuits in the literature is to employ the approximated circuit on an application it would normally be used for and evaluate its performance. This is a popular method because of its simplicity, but the work risks being applicable only to the tested application. Examples of such works [23, 24] have utilized approximate multipliers and adders in image processing scenarios. The error metric then is measured on the test data (input images) and how well the approximate circuits formed the image as opposed to the ideal situation.

Another important implication of approximate circuit fabrication is the acceptance of faulty units that fall within the appropriate range of error metrics required. The authors of [25] presented an approximation-aware test approach to identify approximate-redundant faults. The proposed approach guaranteed that the faults had effects that fall below the acceptable thresholds using a SAT solver and miter approach, and specific automatic test generation patterns.

It can be noted that the most prominent form of error metric evaluation has been the Monte Carlo simulation. This might be a feasible way of determining error metrics for smaller designs. The major issue with the simulation approach is the simulation time that grows exponentially with data width and computation length [14] for evaluating larger approximate designs. The same issue lies with the analytical approaches as they become harder and more complicated to deploy with increased design complexity, particularly if accuracy is considered. The BDD approach can produce exact determinations of error metrics as reported in the works above. In fact, in some approaches this is favorable, since the synthesis procedure requires BDD representation in the first place, and therefore, error metrics can be computed during minimization [16]. Some of the error metrics, such as the error rate, are SAT problems, since they require counting in the solution space [10]. However, SAT approaches to BMC are more scalable than their BDD counterparts [26]. Therefore, the goal of this paper is to implement a SAT solver-based scalable approach in order to enable exact error metrics calculation. The two-error metrics that have been previously implemented using SAT solvers are the worst-case error [15] and bit-flip error [10]. These are simpler metrics to calculate using SAT solvers as they involve searching for a single input assignment in the solution space that leads to maximum value. This paper aims to add to those approaches by introducing an approach for calculating the maximum error rate and an average-case error determination via SAT solving on a CNF representation of an approximated design.

## Preliminaries and background

This section will define glosses over important terminology and key concepts required in this work. The error metrics subsection describes all error metrics, followed by the SAT solver subsection, which describes the main functionality of a SAT solver and recent advancements in the use and applications of SAT solvers. Finally, the miter subsection explains the importance of an auxiliary circuit and how it is used to manipulate designs in order to feed them into a SAT solver and extract a result/metric.

### Error metrics

Approximate circuit designs need to be evaluated to determine their usefulness. One of the important evaluations is how much does the approximated design deviate from the exact one. In the class of functionally-approximated circuits, the approximated circuits are typically compared to the original exact designs to determine the disparity between the two designs. Error rate is a challenging metric to calculate, as it includes checking the probability of error bits overall in the design. Let *f* be a Boolean function that represents the exact design such that *f*: *B*^*n*^→*B*^*m*^ and another Boolean function $\hat{f}:B^{n}\to B^{m}$ represents the approximate circuit. Using this notation, the worst-case error is defined as:

$$e_{wc}(f,\hat{f})=max|int(f(x))-int(\hat{f}(x))|$$ (1)

Eq (1) represents the maximum possible difference between the outputs of the two designs, given that we represent the output of both circuits as integer values [9].

Similarly, bit-flip error can be defined as:

$$e_{bf}(f,\hat{f})=\max\left(\sum_{i=0}^{m-1}\left[f_{i}(x)⊕{\hat{f}}_{i}(x)\right]\right)$$ (2)

Eq (2) denotes bit-flip error, or the maximum number of bits that differ in both outputs.

The error metrics mentioned above have been previously calculated using SAT solver approaches. As noted from Eqs (1) and (2), these error metrics focus on finding a maximum value, which is a single point in the solution space. This makes the problem far more approachable as a binary search technique can be employed along with a specifically designed auxiliary circuit to calculate both metrics. Binary search reduces the computational complexity of the problem.

Error-rate and average-case error are more computationally intensive as they require iteration over all solutions/errors possible. Error rate can be stated as:

$$e_{er}(f,\hat{f})=\frac{\sum_{x\in B^{n}}\left[f(x)\neq \hat{f}(x)\right]}{2^{n}}$$ (3)

This can be read as the fraction of inputs that produce a different output pattern in the approximated circuit other than the exact one [10]. This requires a count of the number of errors between the two circuits (the numerator in Eq (3)) which is also known as the #SAT problem.

Finally, the average-case error can be expressed by:

$$e_{ac}(f,\hat{f})=\frac{\sum_{x\in B^{n}}\left|int(f(x))-int(\hat{f}(x))\right|}{2^{n}}$$ (4)

Eq (4) can be explained as the summation of the absolute difference of outputs of the two circuits produced by every input assignment divided by the number of possible input assignments. Obviously, for the input assignments that do not produce errors, the value inside the summation is zero. Therefore, we need only to perform the summation for values of *x* that produce errors.

### SAT solver

The SAT problem considers a Boolean expression and checks if there is an input assignment(s) that result in the Boolean expression returning a true value. This Boolean expression is input into the SAT solver in the form of a combination of logical clauses. Modern SAT solvers are capable of quickly traversing BDDs and solving millions of propositional clauses in acceptable time frame [5].

SAT solvers have picked up steam as a promising tool to approach many research problems. The applicability of SAT solvers does not just lie in the Boolean network plane. The work of [27] used a SAT-based formulation for calculating the logical-capacity of VIA-configurable structures ASIC (VCSA) fabric. The model used the physical properties of a problem and formulated them into Boolean terms and clauses to be processed by the SAT solver. Another project [28], used a SAT solver to determine short-circuit conditions in logical circuits. Short-circuits in a logic circuit can be induced by several causes such as: a designer connecting two outputs of a module, assuming certain unreliable assumptions. The authors modeled the transistors in the circuit as a graph and tried ensuring that there was no way the power source was connected to the ground via any path. They transformed this problem into a series of Boolean clauses that were then checked by the SAT solver.

There are multiple variations of SAT solving methods (all with the same goal). The details of implementation of such solvers is deemed outside the scope of this paper. However, for the concerned interested reader, information on the SAT solvers relevant to this work is available in [29]. The SAT solver used for this project is a backtracking-based non-blocking SAT solver. Meaning the SAT solver searches for the counter-examples one by one with backtracking without storing the counter examples back into the original problem. This specific strategy was chosen simply because it proved to be the best in practice and used the least amount of memory.

SAT solvers accept a specific format of several Boolean clauses. Therefore, one must change the problem at hand of equivalence checking two circuits (and hence average-case error/error-rate calculation) into a SAT problem. This requires us to first combine both circuits into one in order to feed it into the SAT solver.

### Auxiliary circuits

**Fig 2:**
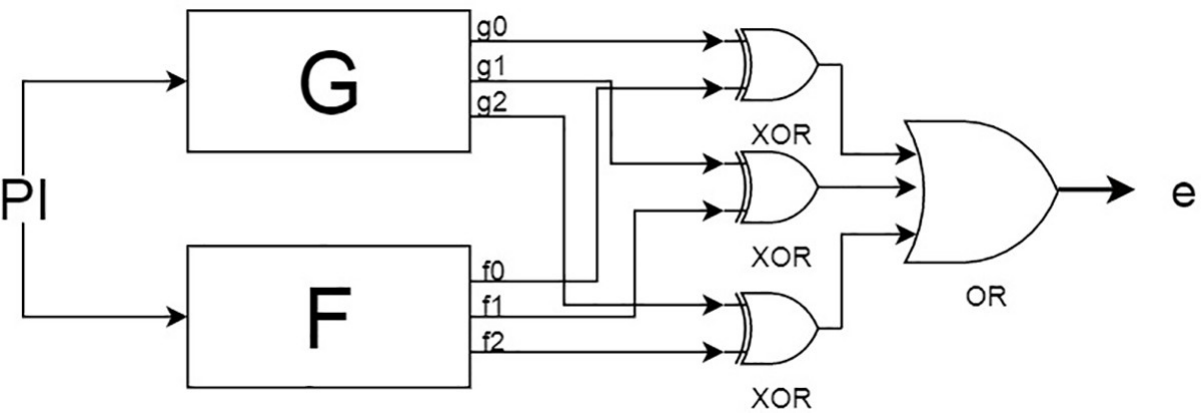
Block diagram of auxiliary circuit.

In order to take advantage of the SAT solver to check equivalence between the two circuits, one must first transform the circuit into a form the SAT solver can use. SAT solvers operate on a number of clauses to determine if they can all be satisfied. Digital circuits can have any number of outputs. Therefore, an auxiliary circuit is built around the exact and approximated designs in order to convert it into a single output and evaluate the desired error metric. Assuming the exact circuit is the black box F and the approximated circuit is the black box G, then the auxiliary circuit can be seen in Fig 2. The general block diagram shows the structure of the auxiliary circuit such that both circuits G and F have the same inputs and both of their outputs lead to the error block, which is the error determination block that produces the error value *e* or assists in finding *e*. The structure of the error determination block differs based on the type of error metric to be determined. The error determination block can simply check if all the outputs from both circuits are equivalent, or it can contain comparators or arithmetic operations to evaluate specific error metric.

## Proposed methodology

**Fig 3:**
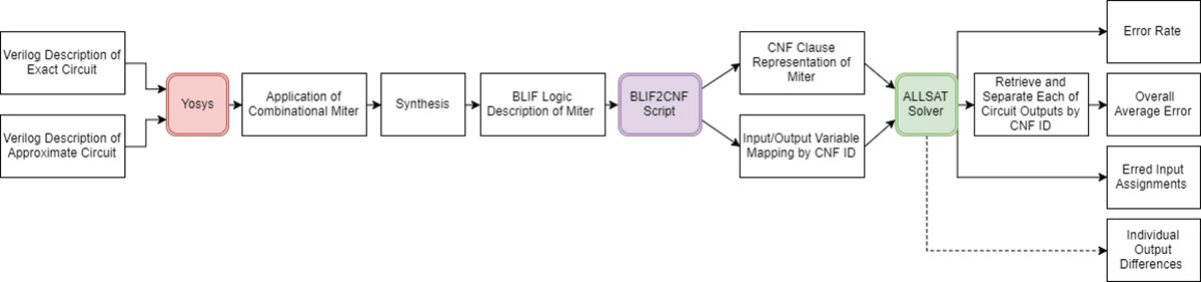
Detailed proposed methodology.

The following subsections present our proposed solutions to the problem of error metrics determination in approximate circuits. The proposed solution consists of three main stages as shown in Fig 3.

Yosys [30] receives the approximate and exact designs, combines them and produces the design as a BLIF (through Yosys). Then, a script processes the BLIF file into a CNF file to enable it to be fed to the SAT solver, and finally, runs the modified ALLSAT solver to determine error metrics for the resulting combined design. Yosys is used to apply the combinational miter, as well as to synthesize the circuit into simple gates/cells. The script is designed to print out a variable mapping that preserves the CNF IDs of the variables of interest that are the inputs and outputs of both circuits. This information is essential to the modified SAT solver to be able to extract the differences between the two circuits and calculate average-case error. The algorithm depends on the fact that both error rate and average-case error require complete coverage of all the errors in the solution space to determine the exact error metric value. Therefore, the SAT solver approach is used to search through the solution space and generate all the error-producing assignments. The number of these assignments can be used to calculate the error rate metric using Eq (2). The input assignments generated can then be simulated for both circuits and used with Eq (4) to calculate average-case error. This form of simulation is cheaper as the run time does not scale with the number of inputs to the circuits. The runtime scales only with the number of unique errors in the circuit. Although the worst possible situation for this approach would be if the number of errors is 2^*n*^ (i.e. every input assignment leads to an error). This is highly unlikely as the circuit usually has a great deal fewer errors than that and synthesis approaches provide an upper limit on number of introduced errors. The modified SAT solver can list the inputs and outputs of the two circuits when errors occur between them. However, listing of the output differences is optional and is not needed for any calculations, but is left as an option for interested designers.

### Auxiliary circuits

The following subsections show examples of the auxiliary circuitry needed to evaluate the maximum error rate and an average-case error. The error rate auxiliary circuit is simply an approximation miter that compares, bit by bit, each of the outputs of the two circuits. In case of the average-case error, some form of accumulator would need to be built around the absolute difference of the two circuits. This is just for clarification and the actual implementation uses an approximation miter for both circuits.

#### Maximum error rate

In the case of error rate, the error determination block is an approximation miter, which is basically ORing the equivalence of each output of both circuits. To illustrate this design concept, we assume a simple design where F and G have three outputs; the structure of the error rate would look like Fig 2. Each output in the circuits F and G are XORed to determine their equivalence. As shown in Fig 2, the outputs (f0,g0), (f1,g1) and (f2,g2) are XORed. Hence, any of these XORed outputs will return a logic 1 wherever their outputs are not equivalent. Therefore, the overall output of the approximation miter will be 1 whenever the output of the approximated circuit G does not match the output of the exact circuit F. The error rate can then be computed as expressed in Eq (3).

Eq (3) represents the global OR of the XORs of each output in both systems (where m is the number of outputs). The number of such inputs that produce a 1 value in the miter are added up and divided by 2^*n*^ to determine the error rate of the approximated system (where n is the number of inputs). Therefore, the addition of the auxiliary circuit turns the two designs into one design with the same number of inputs *n* and one output *e*, where *e* can only be 1 when the two sub-designs are not equivalent [16].

#### Average-case error

**Fig 4:**
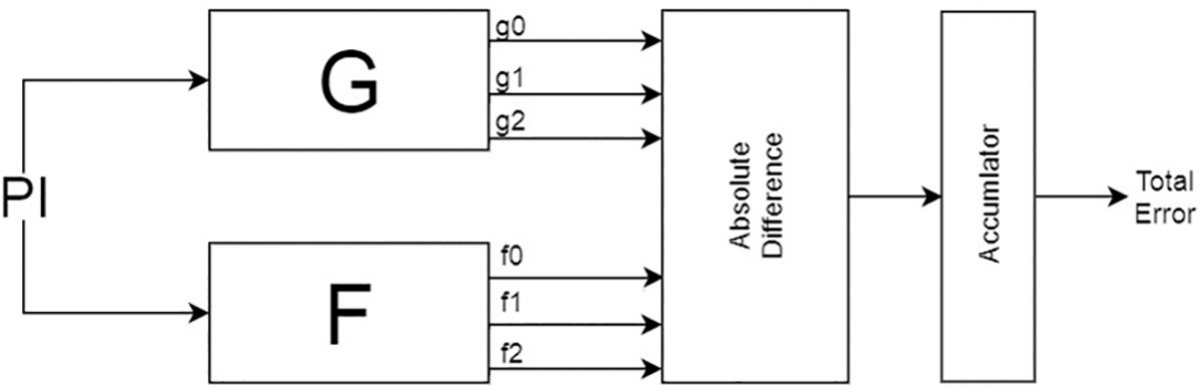
Total error approximation miter example.

Average-case error metric resembles error rate in that it is not concerned with a single output value. Average-case error includes all the possible errors generated by the approximated design. The circuit to calculate the error can be visualized as a combination of the circuits in Figs 2 and 4. The circuit in Fig 2 is used to detect the existence of an error. The circuit in Fig 4 is then used to calculate the value of that error and accumulate it across all input assignments. Therefore, practically the implemented solution is running the SAT solver on the circuit in Fig 2 to enumerate the input assignments that produce errors. Those input assignments are used to create a test bench for the circuit. The average-case error can then be calculated by using Eq (4).

It is important to note is that not all *n* input variables must appear in a solution. The variables that do not appear in a path can be considered do-not cares, as they do not affect the output of the miter. One of the advantages of this process is that it does not need to include every variable and therefore the use of do-not cares can list several input assignments at once and hence the numeration of errors can be summarized. The number of input assignments represented by a path is equal to 2^*c*^ input assignments where c is the number of do-not cares.

### SAT solving

#### Maximum error rate

Error rate involves counting the number of solutions which satisfy the SAT problem. This can be denoted as the ALLSAT problem, which is searching through the solution space for all input assignments that satisfy a circuit. It can be noted that the #SAT problem, which is counting the number of satisfying input assignments, is enough to solve the maximum error rate metric alone. However, in order to aid the algorithm in calculating the average-case error and error rate in one step, we propose the ALLSAT solution. The ALLSAT is an incremental running of the SAT solver in order to collect all counter examples that lead to satisfying the miter. Note that ALLSAT solver stores implications from past iterations in order to speed up future iterations. This gives the algorithm another advantage that enumerates the input assignments that lead to errors, which can be very useful for debugging purposes. We propose an algorithm that combines all the details mentioned to determine the maximum error rate and average case error as shown in Algorithm 1. Furthermore, using the ALLSAT solution for both error-metrics allows us to only use one auxiliary circuit (Fig 2) instead of having to apply both for the two different metrics.

**Algorithm 1: Error Metric Computation Algorithm**

**Input:** Verilog Circuit *F*, Verilog Circuit *G*

**Output:** Maximum Error Rate M*ER*, Average-case Error *AVG*, Set of counterexamples *L*

    1: *M* = ApplyMiter (*F*, *G*)

    2: *MBLIF* = WriteBlif (*M*)

    3: [*CNF*, *MAP*] = BLIF2CNF (*MBLIF*)

    4: [M*ER*, *AVG*, *L*] = MOD_ALLSAT (*CNF*, *MAP*)

                    **Algorithm 1. General Algorithm of Proposed Methodology**

The algorithm that takes both the exact and approximate designs, denoted as Verilog Circuits *F* and *G*, respectively, as indicated in Algorithm 1. The algorithm returns the maximum error rate and average-case error as a result at the function’s termination and has the option to return the set of error-producing input assignments *L*. Line 1 shows a Boolean network constructed from *F* and *G* by combining them in miter *M* and adding the auxiliary circuit shown in Fig 2 using Yosys. The newly-developed Boolean Network M is then written in BLIF format as shown by Line 2. The algorithm calls a script BLIF2CNF in Line 3 to convert this combined BLIF circuit into CNF format to be fed into the modified SAT solver in Line 4. The script turns all variable names into index numbers, and therefore a separate map file of all-important variable indexes is printed. This map file contains the variable indexes for the shared inputs of both combined circuits and the individual outputs of both the exact and approximate circuits. This is useful because the output corresponding to an input can be extracted directly from the counterexample provided by the SAT solver as shown in Algorithm 2. The modified SAT solver MOD-ALLSAT then uses the CNF file and the variable map in order to calculate maximum error rate (MER), average-case error (AVG), and optionally the list of error- producing input assignments (counterexamples) denoted as L.

**Algorithm 2: Modified ALLSAT Algorithm (MOD-ALLSAT)**

**Input:** CNF File *CNF*, Variable I/O Map *MAP*

**Output:** Maximum Error Rate *MER*, Average-case Error *AVG*, Set of counterexamples *L*

    1: *AVG* = 0

    2: ER = 0

    3: **While** *CNF* is satisfiable **do**

    4:     *CEX* = SAT (*CNF*) {*CEX* is a total variable assignment}

    5:     [*input*, *output1*, *output2*] = ExtractRelevantVariables (*CEX*, *MAP*)

    6:     Add input to *L*

    7:     *AVG* = *AVG* + | *int(output1)–int (output2)* |

    8:     *ER ++*

    9:     Update Stored SAT Solving Implications

    10: **end while**

    11: *MER = ER/2*^*n*^

    12: *AVG* = *AVG*/2^n^

                    **Algorithm 2. Modified ALLSAT Solver Algorithm**

A more detailed view of the modified ALLSAT solver (MOD-ALLSAT) used in Algorithm 1 can be seen in Algorithm 2. The MOD-ALLSAT algorithm takes a CNF file and a variable map as inputs and calculates error-rate (ER), average-case error (AVG), and optionally produces the list of counterexamples *L*. The variables *ER* and *AVG* are initialized to zero at the beginning of the algorithm. The ALLSAT solver begins to iteratively find errors in the CNF file. The SAT solver knows that there are no more remaining errors and it can terminate the search, when the problem is no longer satisfiable (Line 3). The SAT solver then works on the CNF file until a satisfying solution (counterexample) is found (Line 4). This solution is presented as a total variable assignment *CEX* to all inputs, outputs and intermediate variables. It is therefore obvious to state that the value of the single output of the miter will be logic 1 for any satisfying variable assignment. However, for our intended purposes, many intermediate variables are not needed, and in fact, we only care about the original inputs and outputs of both circuits. As seen in Line 5, the assignments the SAT solver found to these relevant variables are extracted from the counterexample. The assigned *input* is added to the list of error-producing input assignments (Line 6). The two outputs corresponding to the outputs of the original exact and approximate circuits are also extracted. We consider the absolute difference between the integer representation of each value *output1* and *output2* and accumulate it in the variable *AVG* (Line 7). In Line 8, the variable *ER* is incremented to count error-producing input assignments. The SAT solver then updates its internal information before reiterating the entire process until termination (Line 9). This information may include storing intermediate variable implications it learned to avoid redundant processing, as well as taking note of the input assignment it found in the past loop iteration. This is done in order to eventually eliminate all error-producing input assignments and thus making the problem not satisfiable.

A clarifying analogy to this would be deleting the path that has led to the 1 node in the BDD representation so it cannot be followed again. The SAT solver does this iteratively until eventually the equation cannot be satisfied anymore. This means all errors have been detected. The ALLSAT solver is also special in that it does not start from scratch on every iteration (it learns from previous iterations).

After terminating the search, the variable *ER* now contains the numerator of Eq (3) for maximum error rate calculation. The variable *AVG* also contains the numerator of Eq (4). The error-rate and average-case error can then be computed by dividing both these variables by 2^*n*^, where n is the number of inputs (Lines 11–12). Then the algorithm finishes execution and outputs the error-rate in variable *ER*, average-case error in variable *AVG*, and optionally the list of error-producing inputs *L*. It is worth nothing that both errors are calculated in one go of the SAT solver and with the same auxiliary circuit (the approximation miter).

#### Methodology correctness–an illustrative example

In order to prove the correctness of the designed methodology, a naïve example was developed for testing. A simple exact ripple carry 4-bit adder and an “approximated” 4-bit adder were written in Verilog. The “approximated” adder was a normal ripple carry adder that added one to every value it calculated to artificially introduce errors. For example, in the approximated adder: 1+1 = 3, 2+2 = 5, etc.

Following the steps of Algorithm 1, the inputs to the algorithm are two circuits: an exact one *F* (the ripple carry 4-bit adder), and the approximated circuit (adder that increments valid result by one) *G*. The two circuits are 4-bit adders; therefore, they have 5 outputs each. Yosys is used to apply a miter that XORs each of the 5 outputs to the corresponding output in the other circuit and ORs the result of all the XORs (Line 1). The resulting circuit is produced from Yosys to be converted into a CNF file (Line 2) so it can be fed to the ALLSAT solver. The ALLSAT solver runs on this representation of the miter combining the two circuits (Line 3) and begins to extract all the errors (differences) between them.

Now, since the approximate adder was made to increment one to the result of the exact adder, this means that all input statements produced errors. Hence, the error-rate is 100% (Line 4) and the number of errors (the cardinality of set *L*) is 256 (8 inputs to the miter circuit, 4 per adder). Lastly, the average-case error is calculated by adding each of the differences generated by all input assignments and dividing by the number of possible assignments. Since the approximate circuit simply outputs the result of the exact adder plus one, then the difference in all errors is one. Therefore, the total accumulated difference is 256 (256 errors of value, 1 each), calculated in Line 7 of the algorithm. The average error rate is then 256/256 (Line 9) such as in Eq 4, equaling 1. All numbers mentioned in this example were achieved practically using the proposed methodology and reported by the SAT solver.

## Experimental results

The methodology was implemented using a combination of tools on the library of approximate adders [31]. The Yosys package was used to read two separate Verilog circuit designs (one for the exact and one for the approximated circuit). The Yosys package, with the help from the ABC [32] verification package, was then used to apply the miter circuit, such as the one shown in Fig 2. This design was then synthesized and written into BLIF format. The BLIF format was converted to CNF format (which is a combination of logic clauses) in order to be fed into the SAT solver. This led to a numbering of all variables, which then lose their name when converted to the CNF file. The mapping between the variable names is saved in a map for future processing. The SAT solver used was a modified ALLSAT non-blocking clause solution based on MiniSAT [27]. The solver prints out all input assignments leading to errors and can print out individual error values as well. The solver accumulates all the errors and calculates the average error value. All experiments were carried out on a machine running Linux with an Intel ® core i7 CPU @ 2.9 GHz and 16 GB of memory. Implementation of the described methodology can be obtained at: https://github.com/AliRady/error-metrics-determination/

### Experimental results on benchmark adders

Tables 1 and 2 contain the results of running the proposed methodology on a library of 8-bit and 16-bit approximate adders (which have 16 and 32 effective inputs, respectively). The first column denotes the name of the circuit. The size of the approximated adders in basic cells/gates is reported in the second column. In addition, the size of the merged circuit when applying the miter to the approximate adder and the exact ripple carry adder is reported in the Gates in Miter in the third column. Yosys reported all sizes after synthesizing the circuits into basic logic cells. The total number of errors shown in column 4 is the cardinality of the counter-example set found by the ALLSAT solver, which is then used to calculate maximum error rate using Eq (3). The summation of errors in column 5 is the total sum of the absolute difference between the two circuits over all input assignments, used in average-case error calculation. The tables then contain the results of the two metrics: maximum error rate and average-case error in columns 6 and 7, respectively. The last column represents the processing time over the BLIF file containing the two circuits to be compared. The CPU time with numeration shows the required time to run our methodology including the generation of the file listing the inputs that generates the errors.

**Table 1:** Evaluation of 8-bit approximate adder library.

Approximate Adder	Gates in Approx. Adder	Gates in Miter	Total Number of Errors	Sum of Errors	Max. Error Rate/ Probability	Average-case Error	CPU Time with Numeration (sec)
**ACA_II_N8_Q4**	39	83	12288	491520	18.75	7.5	0.125
**ACA_I_N8_Q5**	52	81	3072	229376	4.67	3.5	0.031
**GDA_St_N8_M4_P2**	39	82	12288	1545024	18.75	23.58	0.138
**GDA_St_N8_M4_P4**	37	64	1536	295488	2.34	4.51	0.011
**GDA_St_N8_M8_P1**	26	94	39424	2064384	60.16	31.5	0.337
**GDA_St_N8_M8_P2**	35	106	19712	2660192	30.08	40.59	0.163
**GDA_St_N8_M8_P3**	45	104	8192	1272896	12.5	19.42	0.116
**GDA_St_N8_M8_P4**	44	117	3072	229376	4.67	3.5	0.034
**GDA_St_N8_M8_P5**	63	123	1024	98304	1.56	1.5	0.010
**GDA_St_N8_M8_P6**	70	131	256	32768	0.39	0.5	0.004
**GeAr_N8_R1_P1**	26	83	39424	5320432	60.16	81.18	0.348
**GeAr_N8_R1_P2**	35	85	19712	3077560	30.08	46.96	0.165
**GeAr_N8_R1_P3**	47	80	8192	491520	12.5	7.5	0.101
**GeAr_N8_R1_P4**	52	74	3072	229376	4.69	3.5	0.034
**GeAr_N8_R1_P5**	43	88	1024	98304	1.56	1.5	0.010
**GeAr_N8_R1_P6**	45	61	256	32768	0.39	0.5	0.0013
**GeAr_N8_R2_P2**	39	81	12288	1553472	18.75	23.70	0.138
**GeAr_N8_R2_P4**	37	75	1536	245440	2.34	3.75	0.009

**Table 2:** Evaluation of 16-bit approximate adder library.

Approximate Adder	Gates in Approx. Adder	Gates in Miter	Total Number of Errors	Sum of Errors (x^10^)	Max. Error Rate /Probability	Average -case Error	CPU Time with Numeration (sec)
**ACA_II_N16_Q4**	75	202	2052587520	879.39	47.79	2047.5	4.435
**ACA_II_N16_Q8**	104	162	251658240	1097.29	5.86	2554.83	1.414
**ACA_I_N16_Q4**	103	226	1462239232	879.39	34.05	2047.5	3.937
**ETAII_N16_Q4**	75	214	2052587520	879.39	47.79	2047.5	4.442
**ETAII_N16_Q8**	104	176	251658240	206.12	5.86	479.91	1.412
**GDA_St_N16_M4_P4**	110	197	251658240	54.76	5.86	127.5	1.421
**GDA_St_N16_M4_P8**	119	201	7864320	0.3221	0.18	7.5	0.473
**GeAr_N16_R2_P4**	81	206	496238592	219.69	11.55	511.5	1.713
**GeAr_N16_R4_P4**	104	192	251658240	54.76	5.86	127.5	1.401
**GeAr_N16_R4_P8**	89	169	7864320	0.3221	0.18	7.5	0.472
**GeAr_N16_R6_P4**	114	168	132120576	13.53	3.08	31.5	0.882

Maximum error rate quantifies the probability that given any two input statements, the outputs of the two adders would be different. The average-case error is a mean value of how large the error would be. For example, there is an 18.75% chance to get an inaccurate output when using the ACA_II_N8_Q4 approximate adder. The expected value of this error (the difference between the two outputs) is 7.5. The main bulk of the CPU time is spent on the SAT solving phase of the methodology, as the application of the miter has negligible processing time. As expected, the CPU time jumps significantly between the 8-bit adders and the 16-bit adders, because the search space grows exponentially with more inputs, making it more compute-intensive to find errors.

The error rate numbers for all the approximate adders exactly matched the ones mentioned in [16], validating that they are correct. The paper did not report an average-case error and therefore the validity of the average-case error results is based on the tests performed with pre-determined results. All numbers reported are exact, therefore achieving them is complex, which is reflected in the runtimes. It is not a challenging task to provide limitations on the metrics, such as terminating the program when a certain error-rate or a certain average-case error is exceeded. Moreover, the authors in [17] did not claim their work to be 100% accurate to the actual error metrics (as they did not provide the values for the errors). In addition, they did not provide all the input combinations that produced errors and they only calculated the metrics. Furthermore, they computed the time for each stage and then calculated each error metric individually. However, our work provides all the input configurations that produce the errors and calculates all error metrics in one run.

From the results, several approximate adders have the same error-rate but different average-case error, such as the ACA_II_N8_Q4 and the GDA_St_N8_M4_P2. The error metrics can therefore be used to categorize circuits into more than one category. Even if the two approximate adders are equally likely to produce an error, the one with a higher average-case error will produce errors that (on average) deviate more from the exact value. This information gives insight to the designer who may value errors that are more impactful over area or power improvements, or vice versa. It is also not impossible for two structurally different adders to have the same value for both metrics, leaving the design choices up to other error metrics or area/power considerations.

**Fig 5:**
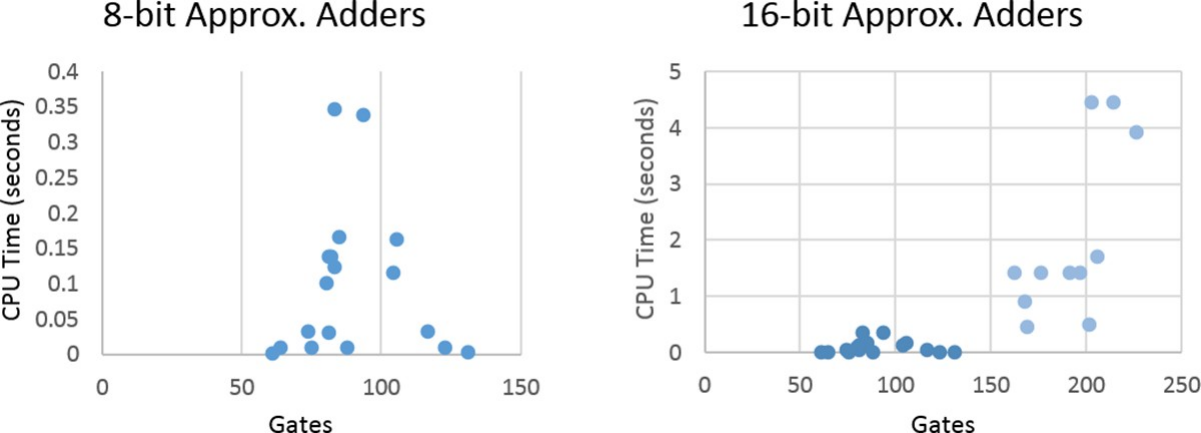
Plots of number of gates in miter vs. CPU time for 8-bit and 16-bit adders.

To discuss the effect of different design parameters on the runtime of the methodology, the CPU time is reported. Firstly, the effect of the miter size in gates/cells on the runtime is explored in Fig 5.

**Fig 6:**
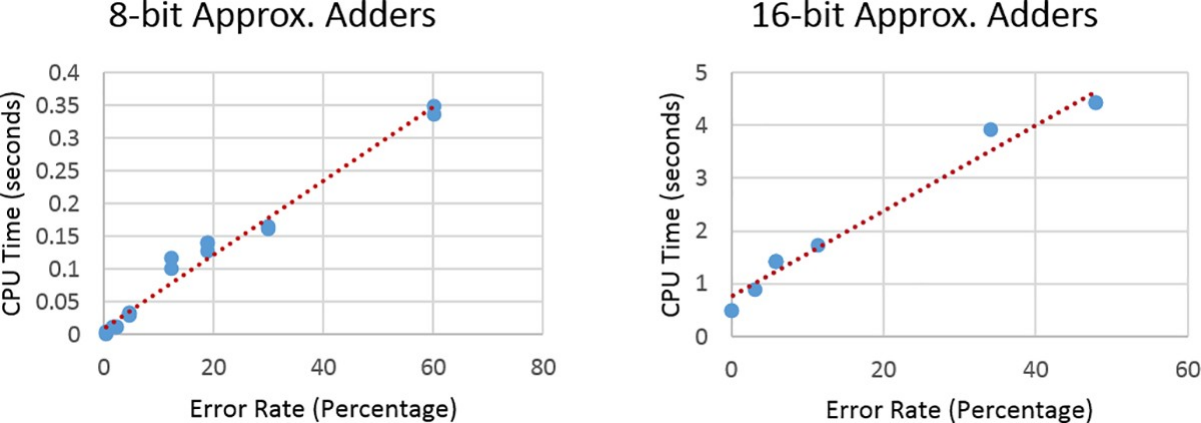
Plots of maximum error rate vs. CPU time for 8-bit and 16-bit adders.

**Fig 7:**
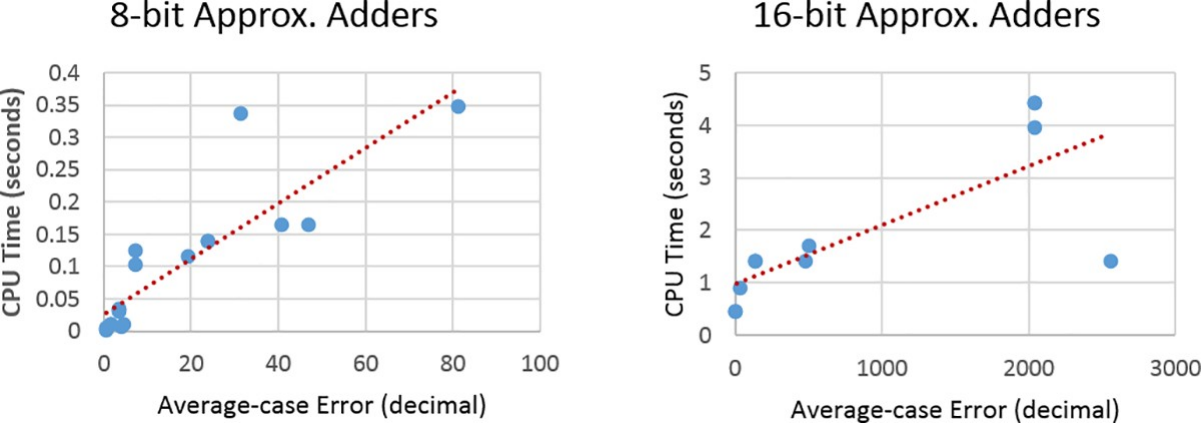
Plots of average-case error vs. CPU time for 8-bit and 16-bit adders.

The first noticeable difference between the two graphs is the jump in CPU time between the 8-bit adders and the 16-bit adders. This is due to the search space doubling with every introduced input variable, leading to an exponential growth. The other noticeable fact is that there is no pattern to the scatter graphs and hence a more influential variable affecting the CPU time. This factor can be demonstrated by looking at Figs 6 and 7. The CPU time is directly proportional to the maximum error rate and an average-case error in the circuit. As the error-rate or average-case error increases, the CPU time increases linearly for both the 8-bit and the 16-bit benchmark sets.

The CPU time scales with the maximum error rate because the higher the maximum error rate the more the number of errors that need to be discovered, therefore more work.

The average-case error metric has more to do with the severity of the error, i.e., the average absolute difference between the two circuits in value when considering both their outputs as integers. For example, two approximate circuits might both have one error. But one of them has the error in the most significant bit and the other in the least significant bit. Therefore, they both end up taking the same CPU time to process but different average-case error.

**Fig 8:**
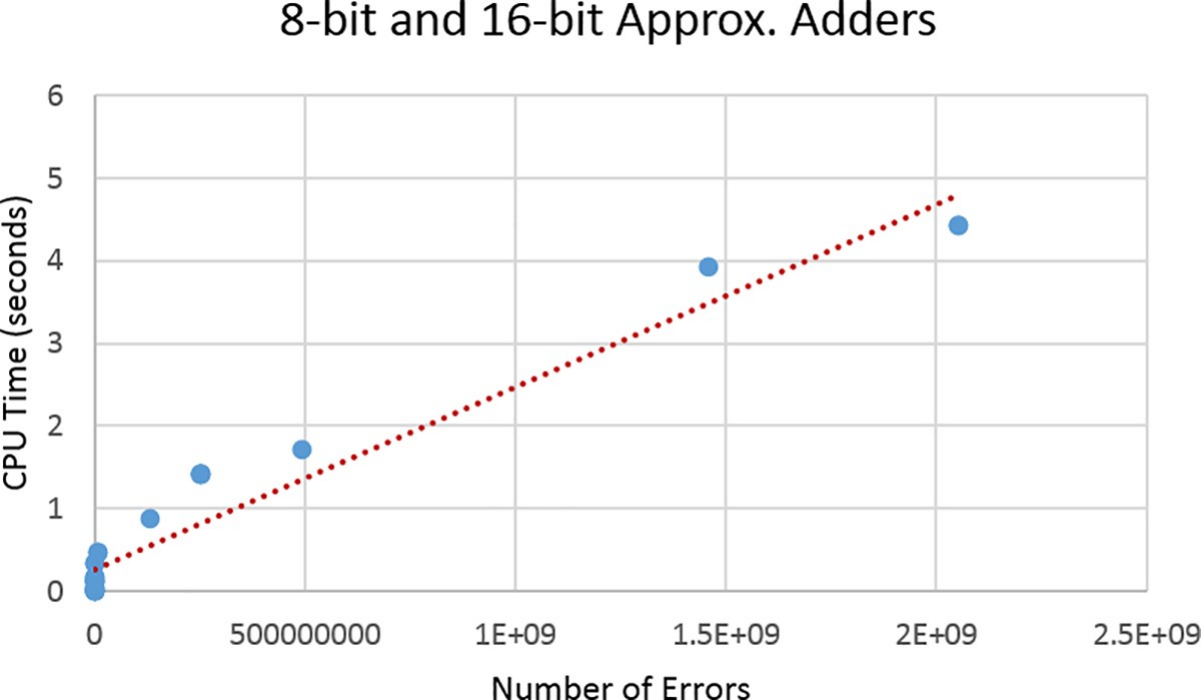
Plots of number of errors vs. CPU time for all benchmarks.

Finally, this effect is consistent even if both benchmarks sets are considered together. However, in order to plot both benchmarks sets together, we need to consider the number of existing errors (Total Number of Errors in Tables 1 and 2) as the size of the search spaces are different. The resulting plot indeed produces a linear relation as shown in Fig 8.

The proposed SAT solving methodology was shown to be applicable to the new error metrics studied by deriving correct error metric results to the benchmark circuits. The approach provided a quantifiable measurement of the two error metrics in one step to exactly determine the difference between the approximate and the correct design. This is done by providing the set of all error-producing assignments and the errors produced by them to determine the practicality of approximate circuits.

## Conclusion and future works

Approximate computing has gained enormous research attention recently by providing a trade-off between computational accuracy and computation effort for the emerging inherent error-tolerant applications. However, to reap the promising benefits of approximate computing, fast and accurate error metrics evaluation methodology is of paramount importance. Error metrics are a crucial factor in the usefulness of approximate circuits in real-life applications. The relevant importance of an error metric is application dependent, and hence, there should be efficient procedures to accurately determine all error metrics. In this paper, two key error metrics were considered that have not been addressed before using a SAT solver methodology. However, the approach is applicable to evaluate all relevant error metrics. In our opinion, this may be the first work to propose SAT solver method for the evaluation of all relevant error metrics in approximate computing. Experimental results using several benchmark approximate adders with varying sizes showed the feasibility of the proposed SAT based methodology. There are some key advantages to the approach, as it provides the set of all error-producing assignments and optionally the errors produced by them. Moreover, it offers a test generation method to facilitate design choices and acts as an important guide to debug the approximate circuits (perhaps there is an error where it should not be). The proposed work was effective in processing the two error metrics in one go, and provided a log of error-generating input assignments in a reasonable timeframe. It was shown experimentally that the processing time of the methodology increases, as expected, rapidly with a few inputs but only linearly with the number of errors it needs to detect.

However, several challenges remain that open interesting research opportunities for future work to realize the full potential of the emergent approximate computing paradigm. It includes the study of the correlation of metrics with each other and with approximation techniques, as well as the evaluation of combined error-metrics and their impact (e.g. Hamming distance) on different applications. Another important extension of this work will be experimenting with the internal algorithm of the SAT solver and discovering if different algorithms might behave better with some circuits and worse with others. The development of parallelized ALLSAT solvers is likely to be a valuable addition in terms of speed of processing.
